# Immunological profiling and treatment patterns of primary Sjögren’s disease patients with concomitant rheumatoid arthritis: a retrospective cohort study

**DOI:** 10.3389/fimmu.2026.1891805

**Published:** 2026-08-03

**Authors:** Yushan Tu, Lei Hou, Yang Zhang, Yue Wang, Peng Yang, Wukai Ma

**Affiliations:** 1 Graduate School of Guizhou University of Traditional Chinese Medicine, Guiyang, China; 2 The Second Affiliated Hospital of Guizhou University of Traditional Chinese Medicine, Medical Clinical Laboratory Center, Guiyang, China; 3 Department of Rheumatology and Immunology, The Second Affiliated Hospital of Guizhou University of Traditional Chinese Medicine, Guiyang, China

**Keywords:** biologic agents, immune profile, overlap syndrome, primary Sjögren’s disease, rheumatoid arthritis, treatment strategy

## Abstract

**Background:**

The coexistence of primary Sjögren’s disease (pSS) and rheumatoid arthritis (RA) is not uncommon, but its immunological characteristics and treatment patterns remain poorly defined. This study aimed to compare the immune profiles and therapeutic strategies between pSS patients with and without RA overlap.

**Methods:**

A total of 148 patients with pSS were retrospectively enrolled, including 89 without RA and 59 with concomitant RA. Demographic characteristics, inflammatory markers, cytokines, lymphocyte subsets, autoantibodies, and treatment regimens were analysed. Logistic regression and ROC analyses were performed to identify markers associated with pSS+RA overlap group.

**Results:**

Compared with the non-RA group, patients in the overlap group had longer disease duration and higher ESR levels. IL-6, IL-17, and IL-8 levels were significantly elevated, accompanied by increased CD4^+^T-cell percentage and CD4/CD8 ratio, but decreased CD19^+^B-cell percentage. Anti-CCP and RF levels were markedly increased in the RA group. Biologic agents were used more frequently, whereas csDMARDs were used less frequently in the overlap group patients. Multivariable analyses identified IL-6, IgM, and CD19^+^B-cell percentage as independently associated with the overlap group. ROC analysis showed that anti-CCP had the highest discriminative ability (AUC = 0.873), while IL-6 demonstrated moderate diagnostic value (AUC = 0.71).

**Conclusion:**

Patients with pSS and concomitant RA exhibit an immune profile characterized by IL-6 pathway activation, T-cell predominance, and altered B-cell responses, closely resembling RA immunopathology. These findings provide insights into autoimmune overlap syndromes and support IL-6 as a potential therapeutic target in this population.

## 1 Introduction

Primary Sjögren’s disease (pSS) is a chronic systemic autoimmune disease characterised by lymphocytic infiltration of the exocrine glands, with clinical manifestations primarily including xerostomia and xerophthalmia. Approximately one-third to one-half of patients may develop extraglandular manifestations involving the joints, skin, lungs, kidneys and nervous system (1, 2). The global prevalence of pSS is approximately 0.05–0.10%, with a strong female predominance (2, 3).

Rheumatoid arthritis (RA) is an autoimmune disease characterised by symmetric polyarthritis, and its typical pathological feature is synovitis, which can lead to joint destruction and functional disability. pSS and RA, both of which belong to the autoimmune disease spectrum, may coexist in the same patient, forming an overlap syndrome. Previous studies have reported that the proportion of pSS patients with concomitant RA ranges from 4% to 31% (4), and these overlap patients often have more severe disease and require more complex treatment. However, the immunological features of this pSS-RA overlap subtype remain poorly defined. Conventionally, pSS is characterised by B cell hyperactivation and production of anti-SSA/SSB antibodies (5), whereas RA is characterised by T cell-mediated synovial inflammation (6, 7) and the production of anti-cyclic citrullinated peptide antibodies (ACPA), most commonly measured as anti-cyclic citrullinated peptide (anti-CCP) antibodies (8).When the two diseases overlap, it remains unclear whether one disease phenotype predominates or an entirely distinct immune profile emerges. Most existing studies have focused on either pSS or RA as single diseases, and systematic immune profiling of the pSS-RA overlap syndrome is lacking. The IL-6/IL-17 axis plays a central role in the pathogenesis of RA, but its role in the pSS-RA overlap syndrome has not been systematically evaluated.

Regarding treatment, the medication patterns for pSS patients with RA overlap are likewise lacking an evidence base. For pSS, sicca symptoms are commonly managed with hydroxychloroquine and symptomatic measures, while glucocorticoids and conventional synthetic disease-modifying antirheumatic drugs (csDMARDs) are used when systemic involvement is present (9). In contrast, RA management emphasises the early use of disease-modifying antirheumatic drugs (DMARDs), including both conventional csDMARDs (e.g., methotrexate) and biologic agents (10, 11). When the two diseases overlap, clinical guidelines provide no clear recommendations on how to choose treatment strategies (12). This knowledge gap leaves clinicians managing pSS-RA overlap patients without evidence-based guidance, often relying on experience rather than evidence. Furthermore, an important and unresolved clinical question is whether pSS-RA overlap patients possess a distinct immunophenotype that leads to differential responses to conventional csDMARDs and biologic agents. This question has direct implications for individualised treatment strategies in this population.

We hypothesized that in pSS patients with concomitant RA, the immune signature is characterized by activation of the IL-6/IL-17 axis, a hallmark of RA immunopathology. However, due to the cross-sectional design and the fact that we enrolled patients from a pSS cohort, we cannot determine whether this signature reflects RA driving the phenotype or a unique pSS-RA overlap subtype. Our aim was to describe the association and generate hypotheses for future research. Therefore, the present study aimed to systematically compare the immunological features and treatment strategies between pSS patients with and without RA overlap using real-world data.

## 2 Methods

### 2.1 Study design and ethics

This was a single-centre, retrospective, real-world study. The study protocol was approved by the Ethics Committee of The Second Affiliated Hospital of Guizhou University of Traditional Chinese Medicine (approval number: LW20260324). Owing to the retrospective nature of the study, the requirement for informed consent from all patients was waived.

### 2.2 Study population

Consecutive patients with pSS who attended the Department of Rheumatology and Immunology of our hospital between January 2021 and December 2025 were enrolled. Inclusion criteria: patients who met the 2016 ACR/EULAR classification criteria for pSS (13). Exclusion criteria: (1) duplicate visits; (2) concomitant other autoimmune diseases, including systemic lupus erythematosus, gouty arthritis, osteoarthritis and systemic sclerosis; (3) concomitant infection or malignancy.

**Figure 1:**
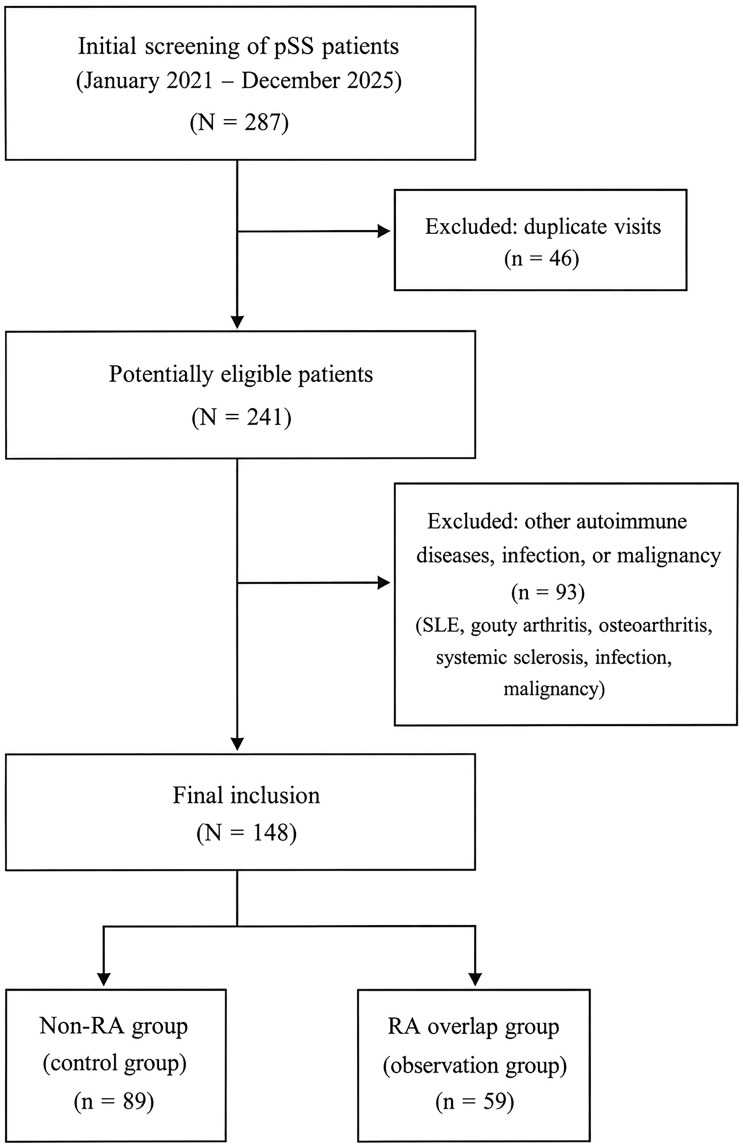
Flowchart of patient enrolment and group assignment. A total of 287 patients with primary Sjögren’s disease (pSS) were initially screened. After excluding 46 duplicate visits and 93 patients with other autoimmune diseases, infection, or malignancy, 148 patients were finally included. Patients were divided into non-RA group (n=89) and RA overlap group (n=59).

According to the above criteria, 287 pSS patients were initially enrolled. After excluding 46 duplicate visits and 93 patients with other autoimmune diseases, infection or malignancy, 148 pSS patients were finally included. Patients were divided into two groups according to the presence or absence of RA: a non-RA group (control group, n=89) and an RA group (observation group, n=59) (**Figure 1**). RA was diagnosed according to the 2010 ACR/EULAR classification criteria for rheumatoid arthritis (14). All patients were recruited from a pSS cohort; thus, the diagnosis of pSS preceded that of RA in all cases. The interval between the two diagnoses was calculated from medical records. It should be noted that patients in the non-RA group may still have other comorbidities (e.g., osteoporosis, thyroid disease, etc.) and are therefore not strictly “pure pSS”. This was an exploratory study, and no prior sample size calculation was performed; the final sample size was determined by the number of consecutive eligible patients during the study period.

Traditional Chinese Medicine (TCM) syndrome types were determined by trained TCM practitioners based on comprehensive evaluation of symptoms, tongue coating, pulse, and other clinical signs. Three categories were used in this study: (1) Cold-Dampness obstruction, characterized by joint pain aggravated by cold or damp weather and a feeling of heaviness; (2) Qi-Yin deficiency, characterized by fatigue, dry mouth, low-grade fever, and shortness of breath; and (3) Others, including mixed or unclassifiable patterns. These syndrome types were included as covariates in multivariable analyses because they influence treatment decisions and clinical management in our local practice.

### 2.3 Data collection

The following information was collected from all patients: demographic and clinical data (age, sex, disease duration, traditional Chinese medicine syndrome type, comorbidities); medications, including glucocorticoids: prednisone, dexamethasone, methylprednisolone, and betamethasone; csDMARDs: Leflunomide, methotrexate, and hydroxychloroquine; biologic agents (including TNF-α inhibitors and IL-6 receptor inhibitors; no patients received rituximab in this cohort); and non-steroidal anti-inflammatory drugs (NSAIDs): diclofenac and celecoxib; laboratory tests (see section 2.4).

### 2.4 Laboratory tests

All blood samples for immunological testing were collected at admission, before the initiation of any new treatment during the current hospitalization. Routine inflammatory markers, including erythrocyte sedimentation rate (ESR), C-reactive protein (CRP), and platelet count (PLT), were measured using standard methods. Immunoglobulins (IgA, IgG, IgM) and complement (C3, C4) were measured using immunoturbidimetry. Lymphocyte subsets (percentages and absolute counts of CD3^+^, CD4^+^, CD8^+^, CD19^+^ and NK cells) were measured using flow cytometry, and the CD4/CD8 ratio was calculated. Cytokines (IL-1β, IL-2, IL-4, IL-5, IL-6, IL-8, IL-10, IL-12P70, IL-17, TNF-α, IFN-α, IFN-γ) were measured using flow cytometric bead array. For autoantibodies, antibodies detected by immunoblotting included anti-SSA/Ro60, anti-SSA/Ro52, anti-SSB/La, anti-nRNP/Sm, anti-Sm, anti-Scl-70, anti-CENP-B, anti-histone H1b, and anti-histone H2b. Immunoturbidimetry was used to measure RF and its isotypes (RF-IgA, RF-IgG, RF-IgM) and anti-CCP. For samples with values exceeding the upper limit of the assay kit, the upper limit value was uniformly used for data analysis.

### 2.5 Statistical analysis

Statistical analysis was performed using SPSS version 26.0 (IBM Corp., Armonk, NY, USA). The Shapiro-Wilk test was used to assess normality of continuous variables. Normally distributed continuous variables are presented as mean ± SD and were compared between groups using the independent-samples t-test; non-normally distributed variables are presented as median (Q_1_, Q^3^) and were compared using the Mann–Whitney U test. Categorical variables are presented as n (%) and were compared using the χ² test or Fisher’s exact test.

To identify immune markers independently associated with RA overlap, all markers that differed significantly between the two groups (ESR, IgM, C3, CD4^+^ T%, CD19^+^ B%, CD4/CD8 ratio, IL-6, IL-8, IL-17) were simultaneously entered into a multivariable logistic regression model using the enter method, with presence of RA as the dependent variable. To examine the robustness of the associations, five sequentially adjusted models were constructed: Model 1 unadjusted; Model 2 adjusted for disease duration; Model 3 further adjusted for TCM syndrome type; Model 4 further adjusted for age; Model 5 further adjusted for biologic agents and csDMARDs. ROC curves were constructed for each marker, the optimal cut-off value was determined using the maximum Youden index, and the area under the curve (AUC) with its 95% confidence interval (CI), sensitivity and specificity were calculated. The proportion of missing data for the main variables was less than <5%; no imputation was performed. A total of more than 30 statistical tests were performed; all analyses were exploratory, and no adjustment for multiple comparisons was applied. Therefore, results with *p*-values close to 0.05 should be interpreted cautiously, and conclusions should primarily rely on effect sizes (OR, AUC, etc.) and the stability of findings with *p* < 0.01. A two-sided significance level was set at *p* < 0.05 (with *p* < 0.001 reported as such).

## 3 Results

### 3.1 Baseline demographic and clinical characteristics

As shown in **Table 1**, the two groups were well-matched in age and sex distribution. However, the overlap group had a significantly longer disease duration (*p* = 0.030) and a distinct TCM syndrome profile (*p* < 0.001), with Cold-Dampness obstruction predominating in the overlap group. Regarding inflammatory markers, ESR was significantly elevated in the overlap group (*p* = 0.021), while CRP and PLT levels were comparable between groups. Among comorbidities, osteoporosis was significantly more common in the overlap group (*p* = 0.003). No significant differences were observed for other comorbidities (all *p*>0.05).

**Table 1:** Baseline demographic and clinical characteristics.

Variable	pSS group (N = 89)	Overlap group (N = 59)	Statistic	*P* value
Demographic characteristics				
Age (years, mean ± SD)	54.19 ± 13.72	57.75 ± 11.01	t=1.741	0.084
Sex, n (%)			Fisher	>0.999
Male	3 (3.37)	2 (3.39)		
Female	86 (96.63)	57 (96.61)		
Ethnicity, n (%)	(N = 89)	(N = 58)	Fisher	0.647
Han	87 (97.75)	56 (96.55)		
Minority	2 (2.25)	2 (3.45)		
Clinical characteristics				
Disease duration (months), median (Q_1_, Q^3^)	24.00 (12.00, 60.00)	46.00 (12.00, 108.00)	U=2075	**0.030**
TCM syndrome type, n (%)	(N = 89)	(N = 58)	χ²=51.89	**<0.001**
Cold-Dampness obstruction	6 (6.74)	29 (50.00)		
Qi-Yin deficiency	55 (61.80)	7 (12.07)		
Others	28 (31.46)	22 (37.93)		
Inflammatory markers				
ESR (mm/h), median (Q_1_, Q^3^)	15.50 (8.00, 30.00) (N = 88)	21.00 (14.00, 45.00)	U=2013	**0.021**
CRP (mg/L), median (Q_1_, Q^3^)	1.69 (0.80, 4.77) (N = 84)	3.29 (1.26, 8.59)	U=2017	0.058
PLT (×10^9^/L), median (Q_1_, Q^3^)	196.0 (166.00, 233.00) (N = 87)	201.5 (173.30, 261.30) (N = 58)	U=2201	0.194
Comorbidities, n (%)				
Hypertension	18 (20.22)	12 (20.69)	χ²=0.005	0.946
Osteoporosis	9 (10.11)	17 (29.31)	χ²=8.890	**0.003**
Interstitial lung disease/pulmonary fibrosis	9 (10.00)	6 (10.34)	χ²=0.002	0.964
Thyroid disease	10 (11.24)	8 (13.79)	χ²=0.214	0.644
Chronic gastritis	10 (11.24)	6 (10.34)	χ²=0.029	0.865
Sleep disorder	4 (4.49)	0 (0)	Fisher	0.154
Other comorbidities	36 (40.45)	21 (36.21)	χ²=0.266	0.606

Data are presented as mean ± SD, median (Q_1_, Q^3^), or n (%). *p* values were calculated using the independent-samples t test, Mann-Whitney U test, chi-square test, or Fisher’s exact test as appropriate. ESR, erythrocyte sedimentation rate (an acute-phase reactant/inflammatory marker); CRP, C-reactive protein; PLT, platelet count; TCM, traditional Chinese medicine. Bold values indicate statistically significant differences (P < 0.05).

In addition to the baseline characteristics shown in **Table 1**, we reviewed the medical records of the overlap group to determine the temporal relationship between the two diagnoses. The median interval between pSS diagnosis and RA diagnosis was 18 months (IQR 6–36 months). Regarding radiographic findings, 28 of 59 (47.5%) patients in the overlap group had evidence of erosive changes on plain radiographs or musculoskeletal ultrasound, indicating a substantial burden of joint damage in this subgroup.

### 3.2 Comparison of treatment strategies

As shown in **Table 2**, glucocorticoid use was similar between the two groups (*p* = 0.923). However, the overlap group had significantly higher use of biologics (*p* = 0.002) and significantly lower use of csDMARDs (*p* = 0.001). Among csDMARDs, combination regimens were more common in the pSS group, with methotrexate plus hydroxychloroquine being the most frequently used, while the overlap group exclusively received combination therapy. Hydroxychloroquine alone and hydroxychloroquine plus leflunomide were used only in the pSS group. NSAID use was comparable between groups (*p* = 0.165).

**Table 2:** Comparison of treatment strategies.

Variable	pSS group (N = 89)	overlap group (N = 59)	Statistic	*p* value
Glucocorticoids, n (%)				
Yes	67 (75.28)	44 (74.58)	χ²=0.009	0.923
No	22 (24.72)	15 (25.42)		
Biologics, n (%)				
Yes	8 (8.99)	17 (28.81)	χ²=9.933	**0.002**
No	81 (91.01)	42 (71.19)		
csDMARDs, n (%)				
Hydroxychloroquine + Methotrexate	17 (19.10)	2 (3.39)	Fisher	0.005
Hydroxychloroquine alone	2 (2.25)	0 (0.00)	Fisher	0.517
Hydroxychloroquine +Leflunomide	2 (2.25)	0 (0.00)	Fisher	0.517
Total	21 (23.60)	2 (3.39)	Fisher	0.001
NSAIDs, n (%)				
Yes	6 (6.74)	8 (13.56)	χ²=1.926	0.165
No	83 (93.26)	51 (86.44)		

Data are presented as n (%). *p* values were calculated using the chi-square test or Fisher’s exact test as appropriate. csDMARDs, conventional synthetic disease-modifying antirheumatic drugs; NSAIDs, non-steroidal anti-inflammatory drugs. Individual csDMARD regimens are listed in the table; total csDMARD use is shown in the ‘Total’ row. Bold values indicate statistically significant differences (P < 0.05).

### 3.3 Comparison of autoantibodies and complement

As shown in **Table 3**, the overlap group had significantly higher levels of RF, RF-IgA, RF-IgM, and anti-CCP compared with the pSS group (all *p* < 0.001), whereas RF-IgG levels did not differ significantly (*p* = 0.094). Complement C3 was significantly elevated in the overlap group (*p* < 0.001), while C4 levels were comparable between groups (*p* = 0.433).Positive rates of pSS-associated antibodies (anti-SSA/60kd, anti-SSA/52kd, anti-SSB/La) did not differ between groups (all *p*>0.05). The positive rate of anti-hxth2xb was significantly higher in the overlap group (52.94% vs. 26.56%, *p* = 0.004), while anti-histone H1b showed no intergroup difference (*p*>0.999). Antibodies with low positive rates (<6%, including anti-Scl-70, anti-CENP-B and anti-Sm) are presented in **Supplementary Table 1**.

**Table 3:** Comparison of autoantibodies and complement.

Variable	pSS group	Overlap group	Statistic	*P* value
RA-associated antibodies, M (Q_1_, Q^3^)	(N = 83)	(N = 55)		
RF (IU/mL)	12.77 (7.04, 19.45)	58.84 (16.50, 246.4)	U=859	<0.001
RF-IgA (IU/mL)	5.84 (3.66, 13.48)	22.16 (10.90, 70.40)	U=962	<0.001
RF-IgG (IU/mL)	3.07 (2.16, 4.81)	3.67 (2.39, 7.55)	U=1898	0.094
RF-IgM (IU/mL)	15.13 (7.77, 22.91)	53.01 (15.97, 374.20)	U=1072	<0.001
Anti-CCP (U/mL)	3.29 (2.22, 5.81)	331.4 (13.96, 400.00)	U=562.5	<0.001
pSS-associated antibodies, n (%)	(N = 83)	(N = 56)		
Anti-SSA/60kd positive	63 (75.90)	40 (71.43)	χ²=0.348	0.555
Anti-SSA/52kd positive	52 (62.65)	37 (66.07)	χ²=0.170	0.680
Anti-SSB/La positive	21 (25.30)	15 (26.79)	χ²=0.038	0.845
SLE-associated antibodies, n (%)	(N = 83)	(N = 56)		
Anti-nRNP/Sm positive	8 (9.64)	6 (10.71)	χ²=0.043	0.836
Other autoantibodies, n (%)	(N = 64)	(N = 51)		
Anti-histone H1b positive	2 (3.13)	2 (3.92)	Fisher	>0.999
Anti-histone H2b positive	17 (26.56)	27 (52.94)	χ²=8.361	0.004
Complement, M (Q_1_, Q^3^)	(N = 86)	(N = 54)		
C3 (g/L)	1.10 (0.96, 1.20)	1.26 (1.09, 1.37)	U=1540	<0.001
C4 (g/L)	0.26 (0.21, 0.33)	0.26 (0.22, 0.34)	U=2098	0.433

Data are presented as median (Q_1_, Q^3^) or n (%). p values were calculated using the Mann-Whitney U test, chi-square test, or Fisher’s exact test as appropriate. Sample sizes vary due to the availability of laboratory data; not all tests were performed for every patient. M, median; Q_1_, Q^3^, first and third quartiles; RF, rheumatoid factor; anti-CCP, anti-cyclic citrullinated peptide antibodies; SLE, systemic lupus erythematosus.

### 3.4 Comparison of lymphocyte subsets and cytokine profiles

**Figure 2:**
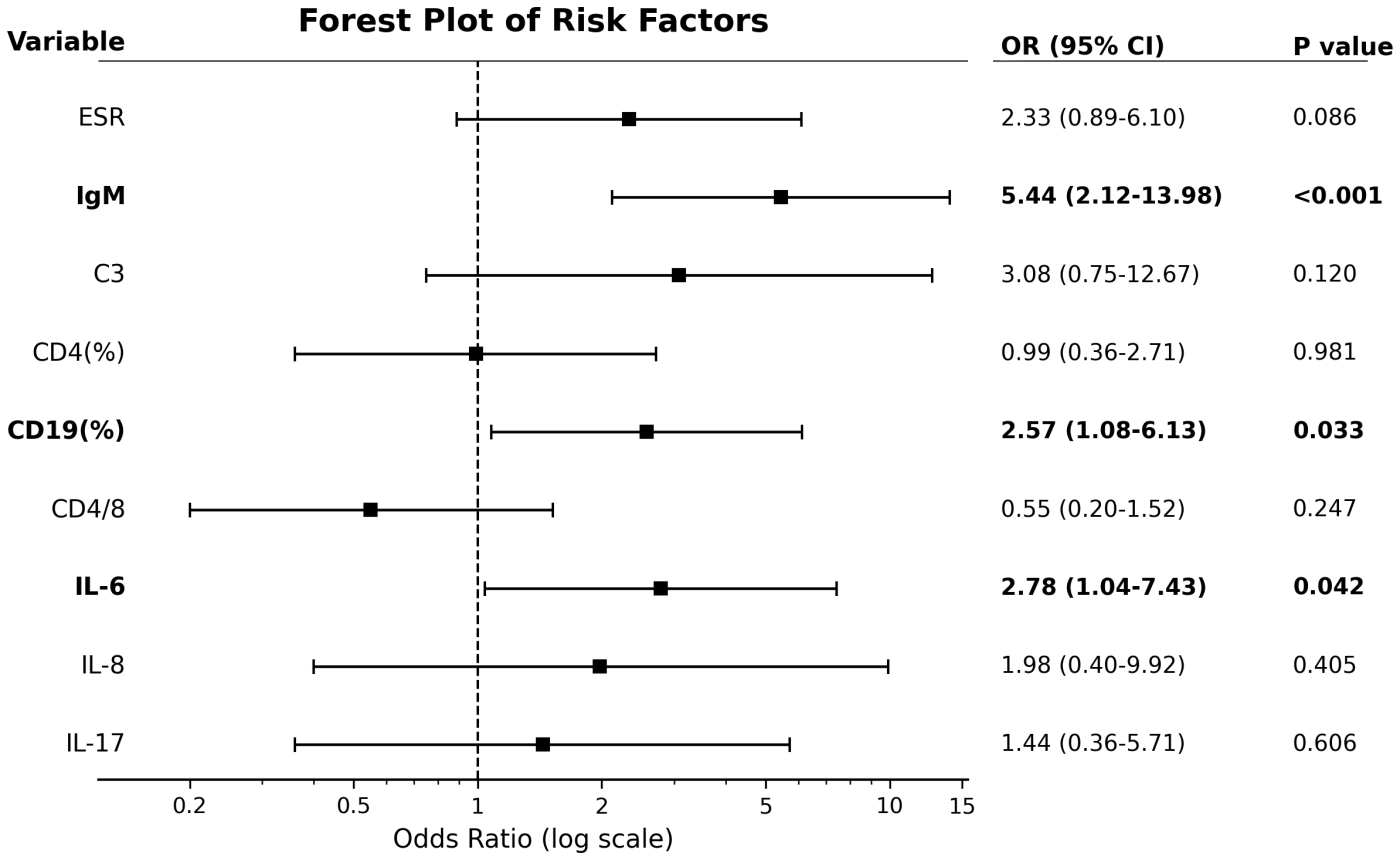
Multivariable logistic regression forest plot of immune markers comparing overlap group vs. pSS. The figure shows odds ratios (ORs) with 95% confidence intervals (CIs) for immune markers significantly associated with overlap group. All markers that differed significantly between the two groups (ESR, IgM, C3, CD4^+^T%, CD19^+^B%, CD4/CD8 ratio, IL-6, IL-8, IL-17) were simultaneously entered into a multivariable logistic regression model using the enter method, with presence of RA as the dependent variable. IgM (OR = 5.44, 95% CI: 2.12–13.98, *p* < 0.001) and CD19^+^B cell percentage (OR = 2.57, 95% CI: 1.08–6.13, *p* = 0.033) were positively associated with overlap group, while IL-6 (OR = 2.78, 95% CI: 1.04–7.43, *p* = 0.042) also showed a significant positive association.

As shown in **Table 4**, CD3^+^T cell percentages did not differ between groups (*p* = 0.567). Compared with the pSS group, the overlap group had significantly higher CD4^+^Th cell percentage (*p* = 0.006) and CD4^+^/CD8^+^ ratio (*p* = 0.012), while CD19^+^B cell percentage was significantly lower (*p* = 0.001). CD8^+^T cell percentage did not differ (*p* = 0.097). Regarding cytokines, the overlap group showed significantly higher levels of IL-6 (*p* < 0.001), IL-17 (*p* = 0.008) and IL-8 (*p* = 0.023). No significant differences were observed for TNF-α, IL-10, IFN-γ, IL-2 or IL-4 (all *p*>0.05), suggesting selective activation of the inflammatory cytokine profile rather than broad systemic inflammation. Although IL-17 showed significant elevation in univariable analysis (*p* = 0.008), it did not retain independent significance in the multivariable logistic regression model (*p*>0.05; **Figure 2**), suggesting that its effect may be mediated through or confounded by IL-6.

**Table 4:** Comparison of lymphocyte subsets and cytokine profiles.

Variable	pSS group	Overlap group	Statistic	*p* value
Lymphocyte subsets (%)	(N = 82)	(N = 56)		
CD3^+^ T cells	70.86 ± 7.36	71.72 ± 9.49	t=0.5745	0.567
CD4^+^ Th cells	40.33 ± 7.68	44.50 ± 9.05	t=2.818	0.006
CD8^+^ cells	25.78 (20.89, 31.94)	24.20 (18.77, 30.47)	U=1913	0.097
CD4^+^/CD8^+^ ratio	1.48 (1.09, 2.16)	1.95 (1.35, 2.64)	U=1720	0.012
CD19^+^ B cells	14.24 (11.45, 21.57)	11.44 (7.74, 16.58)	U=1543	0.001
Th17 axis-related cytokines (pg/mL)	(N = 83)	(N = 56)		
IL-6	1.58 (0.58, 4.30)	5.00 (1.89, 15.43)	U=1367	<0.001
IL-17	1.77 (0.61, 2.14)	1.77 (1.77, 4.03)	U=1722	0.008
IL-8	1.84 (0.92, 2.00)	2.00 (1.73, 2.05)	U=1811	0.023
Control cytokines (pg/mL)	(N = 83)	(N = 56)		
TNF-α	0.22 (0.22, 1.44)	0.40 (0.22, 1.43)	U=2215	0.632
IL-10	0.86 (0.27, 2.30)	0.82 (0.27, 1.63)	U=2317	0.975
IFN-γ	2.24 (0.49, 5.45)	2.31 (1.61, 5.52)	U=2129	0.404
IL-2	0.92 (0.39, 1.59)	1.07 (0.77, 1.67)	U=1965	0.123
IL-4	0.24 (0.24, 1.17)	0.56 (0.24, 0.92)	U=2044	0.223

Data are presented as mean ± SD for normally distributed variables (CD3^+^, CD4^+^) or median (Q_1_,Q^3^) for non-normally distributed variables. Complete data on absolute lymphocyte counts and other cytokines (IL-1β, IL-5, IL-12P70, IFN-α) are provided in **Supplementary Table 2**.

### 3.5 Multivariable logistic regression analysis

In multivariable logistic regression analysis (**Figure 2**), IgM (OR = 5.44, 95% CI: 2.12–13.98, *p* < 0.001) and CD19^+^B-cell percentage (OR = 2.57, 95% CI: 1.08–6.13, *p* = 0.033) were positively associated with the overlap group. IL-6 also showed a significant independent association (OR = 2.78, 95% CI: 1.04–7.43, *p* = 0.042). The remaining markers, including ESR, C3, CD4^+^ T%, CD4/CD8 ratio, IL-8, and IL-17, did not retain independent significance (all *p*>0.05).

### 3.6 Multivariable stratified logistic regression analysis

**Figure 3:**
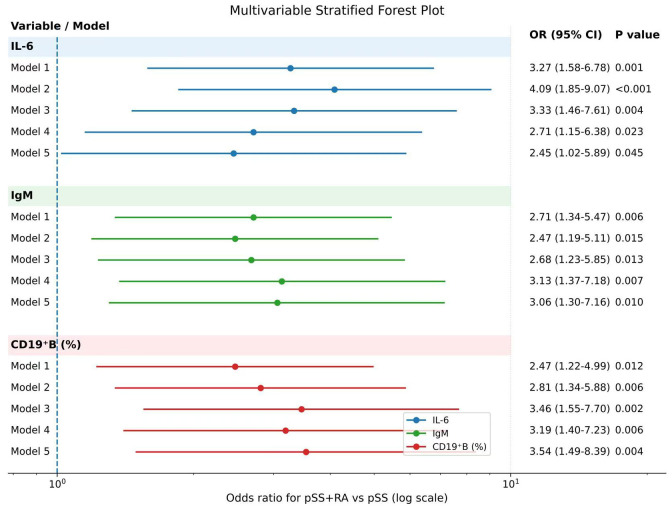
Multivariable stratified logistic regression forest plot of key immune markers comparing overlap group vs. pSS across five adjustment models. Five sequentially adjusted models were constructed to examine the robustness of the associations. Model 1: unadjusted; Model 2: adjusted for disease duration; Model 3: further adjusted for TCM syndrome type; Model 4: further adjusted for age; Model 5: further adjusted for biologic agents and csDMARDs. IL-6, IgM and CD19^+^B cell percentage remained significantly associated with overlap group across all five models (all *p* < 0.05). IL-6 showed stable ORs ranging from 2.47 (95% CI: 1.19–5.11) to 3.14 (95% CI: 1.37–7.18). IgM ORs ranged from 2.71 (95% CI: 1.34–5.47) to 3.06 (95% CI: 1.30–7.16). CD19^+^B cell percentage ORs ranged from 2.47 (95% CI: 1.22–4.99) to 3.54 (95% CI: 1.49–8.39). Squares represent point estimates, and horizontal lines represent 95% CIs.

In sequentially adjusted logistic regression models (**Figure 3**), IL-6, IgM, and CD19^+^B-cell percentage remained significantly associated with the overlap group across all five models (all *p* < 0.05). IL-6 showed stable ORs ranging from 2.45 (95% CI: 1.02–5.89) to 4.09 (95% CI: 1.85–9.07). IgM ORs ranged from 2.47 (95% CI: 1.19–5.11) to 3.14 (95% CI: 1.37–7.18), and CD19^+^B-cell percentage ORs ranged from 2.47 (95% CI: 1.22–4.99) to 3.54 (95% CI: 1.49–8.39), with the strongest effect observed in the fully adjusted model (Model 5). All confidence intervals excluded 1, supporting the robustness of these associations. These findings indicate that the observed differences are independent of disease duration, TCM syndrome type, age, and treatment strategies.

### 3.7 ROC curve analysis

**Figure 4:**
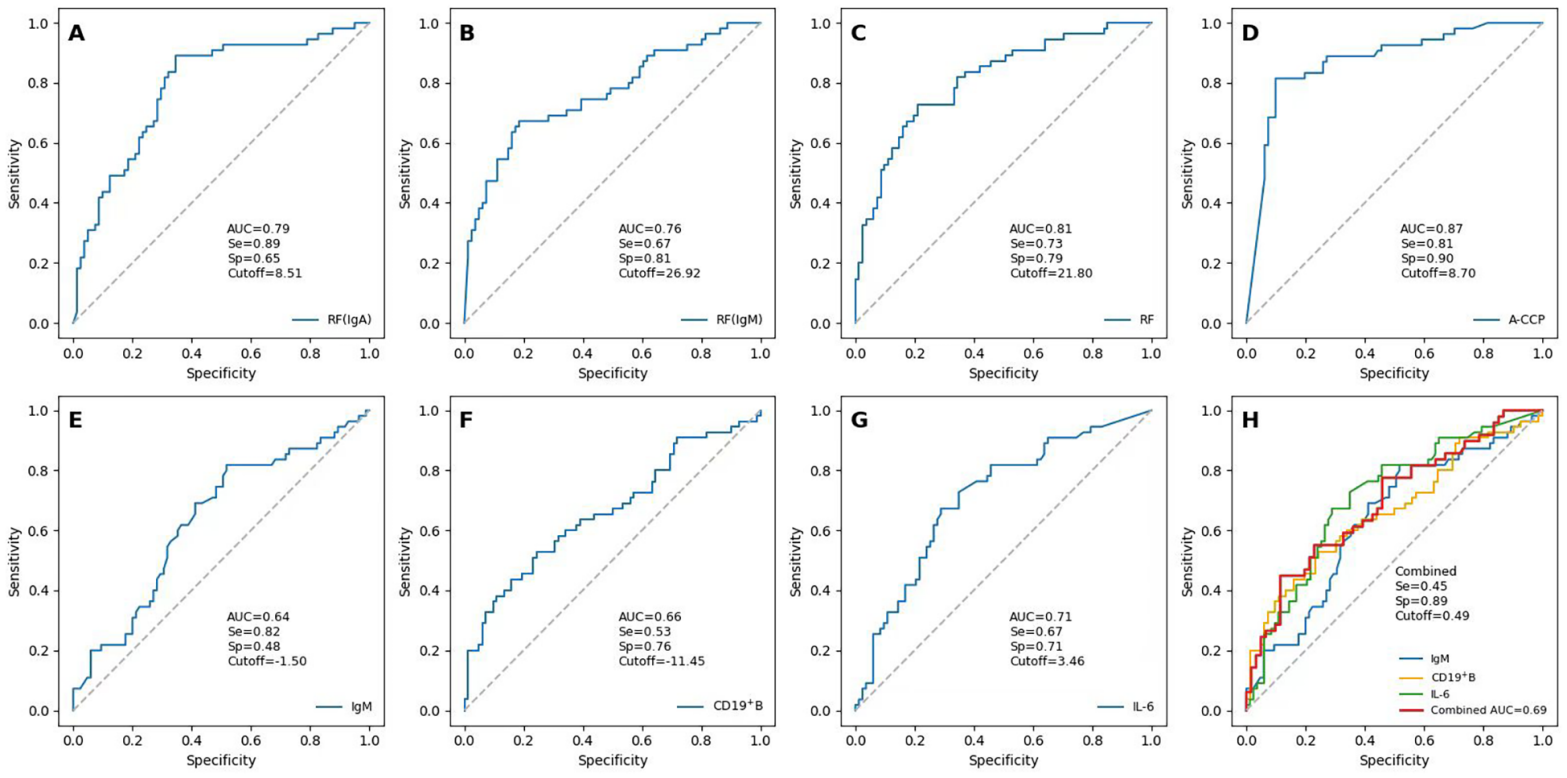
ROC curve analysis: Discriminative performance of traditional and novel immune markers for distinguishing overlap group from pSS. ROC curves were constructed with presence of RA as the dependent variable. **(A)** RF-IgA; **(B)** RF-IgM; **(C)** RF and anti-CCP (anti-CCP AUC = 0.87, RF AUC = 0.81); **(D)** IgM (cut-off 1.50 g/L, sensitivity 0.82, specificity 0.48); **(E)** CD19^+^B cell percentage (cut-off 11.45%, sensitivity 0.53, specificity 0.76); **(F)** IL-6 (cut-off 3.46 pg/mL, sensitivity 0.67, specificity 0.71); **(G)** Combined diagnosis of novel markers (IgM+CD19^+^B%+IL-6, AUC = 0.69); **(H)** Comparison of ROC curves between traditional and novel markers. The optimal cut-off value was determined using the maximum Youden index. AUC, area under the curve.

ROC curves were constructed to evaluate the discriminative performance of traditional and novel immune markers (**Figure 4**). Among traditional RA markers, anti-CCP showed the highest AUC (0.87), followed by RF (0.81), RF-IgA (0.79) and RF-IgM (0.76) (**Figures 4A–D**). Among novel markers, IL-6 yielded an AUC of 0.71 (cutoff 3.46 pg/mL, sensitivity 0.67, specificity 0.71); IgM yielded an AUC of 0.64 (cutoff 1.50 g/L, sensitivity 0.82, specificity 0.48); and CD19^+^ B cell percentage yielded an AUC of 0.66 (cutoff 11.45%, sensitivity 0.53, specificity 0.76) (**Figures 4E–G**). The combination of novel markers produced an AUC of 0.69, slightly lower than IL-6 alone (**Figure 4H**). Overall, anti-CCP demonstrated optimal discriminative ability, while IL-6 showed moderate potential; IgM and CD19^+^B cell percentage showed relatively limited discriminative performance.

## 4 Discussion

In this retrospective cohort study of 148 pSS patients, we found that those with concomitant RA present an immunological signature that closely mirrors core RA immunopathology, characterized by IL-6 pathway activation, T-cell activation, relative B-cell suppression, and marked elevation of anti-CCP and RF. Regarding treatment strategies, glucocorticoid use was similar between the two groups, but patients in the overlap group received fewer conventional csDMARDs and more biologic agents. It is important to note that, because our study was conducted in a pSS patient cohort, we cannot establish whether RA drives this signature or whether it represents a distinct pSS-RA overlap subtype. Nevertheless, the close alignment with RA immunopathology suggests that RA pathology may be a major contributor to the observed immune phenotype, although this interpretation warrants confirmation in future studies.

IL-6, IL-17 and IL-8 levels were significantly higher in the overlap group than in the pSS alone group, with the most marked difference observed for IL-6 (median 5.00 vs. 1.58 pg/mL, *p* < 0.001). IL-6 is a pleiotropic cytokine that plays a central role in RA pathogenesis, promoting B cell differentiation into plasma cells, inducing Th17 cell differentiation, stimulating acute-phase protein synthesis in hepatocytes, and participating in osteoclast activation and joint erosion (15–17). Complement C3 was also elevated in the overlap group (1.26 vs. 1.10 g/L, *p* = 0.0007), while CRP showed a non-significant trend towards elevation (*p* = 0.058). This selective acute-phase response pattern may reflect differential hepatic regulation by IL-6. IL-17, a key effector of Th17 cells, recruits neutrophils, promotes matrix metalloproteinase production by fibroblasts and chondrocytes, and mediates joint cartilage and bone destruction (15, 18). IL-17 levels were significantly elevated in the overlap group (*p* = 0.008), consistent with the articular inflammatory phenotype of RA. Notably, no significant differences were observed in IL-2, IL-4, IL-10 or IFN-γ between the two groups, suggesting that the inflammatory features of the overlap group are selective rather than reflecting broad systemic inflammatory activation. Although IL-17 showed significant elevation in univariable analysis, it did not retain independent significance in the multivariable logistic regression model, suggesting that its effect may be mediated through or confounded by IL-6. This is consistent with the hierarchical organization of the IL-6/Th17 axis, in which IL-6 serves as a critical upstream inducer of Th17 polarization (20). Previous studies have rarely focused on the IL-6/IL-17 axis in overlap syndromes; only one small study (n=32) reported elevated IL-6 in pSS-RA patients (19, 21), but did not measure IL-17. Another study in pSS found that IL-17 correlated with the severity of sicca symptoms, but did not specifically analyse the RA-overlap subgroup (22). Based on these findings, we propose that IL-6, rather than IL-17, is the dominant driver of the inflammatory phenotype in pSS-RA overlap. Importantly, while IL-17 inhibitors have shown inconsistent efficacy in RA clinical trials—with some failing to meet primary endpoints in phase III studies (23, 24)—IL-6 receptor blockade (e.g., tocilizumab) has demonstrated consistent efficacy in RA (25). Therefore, the IL-6 pathway represents a more rational therapeutic target for pSS-RA overlap patients, although direct evidence in this population is still lacking.

Compared to the pSS alone group, the overlap group exhibited a distinct lymphocyte profile, characterized by a higher CD4^+^T cell percentage (44.50% vs. 40.33%, *p* = 0.006) and CD4/CD8 ratio (1.95 vs. 1.48, *p* = 0.012), but a lower CD19^+^B cell percentage (11.44% vs. 14.24%, *p* = 0.001). This pattern sharply contrasts with the classical immunological features of pSS, which are characterised by B cell hyperactivation, often presenting with B cell expansion, hypergammaglobulinaemia, and anti-SSA/SSB antibodies in peripheral blood (26, 27). However, in the present study, the overlap group exhibited a decreased B cell percentage and lower IgM levels (1.14 vs. 1.43 g/L, *p* = 0.007), while IgA and IgG showed no significant differences. Several explanations for this phenomenon are plausible. First, the effect of biologic agents—although no patients in this cohort received rituximab (an anti-CD20- depleting antibody), the use of other biologics (TNF-α inhibitors and IL-6 receptor inhibitors) may still have influenced the immune profile (28); second, the dominance of RA pathology, as RA is primarily T cell-mediated (29); therefore, the concurrent presence of RA shifts the overall immune landscape from a B cell-dominant pattern (typical of pSS alone) to a T cell-dominant pattern; third, B cell trafficking to inflamed tissues, where B cells may preferentially migrate to target organs such as the salivary glands and synovium, leading to their reduced numbers in peripheral blood (30). Consistent with this T cell-dominant shift, the overlap group showed a significantly elevated CD4/CD8 ratio compared to the pSS alone group (1.95 vs. 1.48, *p* = 0.012). An elevated CD4/CD8 ratio is a recognized indicator of immune dysregulation in various autoimmune diseases (31). We therefore propose that the CD4/CD8 ratio could serve as a simple and readily available biomarker for assessing immune dysregulation in patients with pSS-RA overlap syndrome. Importantly, no patients in this cohort had received rituximab, excluding the possibility that the observed B-cell reduction was driven by anti-CD20 treatment. Furthermore, the association between CD19^+^B-cell percentage and the overlap group remained significant after adjustment for biologic agents and csDMARDs in multivariable analysis (Model 5: OR = 3.54, 95% CI: 1.49–8.39). These findings support that the relative B-cell suppression reflects disease biology rather than treatment artifact.

One of the most striking findings of this study was the extremely elevated anti-CCP levels in the overlap group(median 331.4 vs. 3.29 U/mL, *p* < 0.001), representing an approximately a 100-fold difference. RF levels were also significantly higher (58.8 vs. 12.8 U/mL, *p* < 0.001). Such a magnitude of difference is rare in clinical biomarker studies. anti-CCP is a highly specific serological marker for RA and is typically not elevated, or only mildly so, in pSS alone (32). In the present study, the median anti-CCP level in the pSS group was 3.29 U/mL, with the vast majority of patients within the normal range. This large difference strongly suggests that elevated anti-CCP in a pSS patient should be considered strong evidence of concomitant RA rather than a manifestation of pSS itself. Previous studies have reported an anti-CCP positivity rate of approximately 3–10% in pSS patients, and that anti-CCP-positive patients often have erosive arthritis (32). The present study extends this knowledge by showing that the level of anti-CCP may be a key discriminator between pSS and pSS-RA. We suggest that when anti-CCP levels exceed two to three times the upper limit of normal in a pSS patient, concomitant RA should be suspected. RF is also frequently positive in pSS (approximately 30–50%) but has lower specificity than anti-CCP (33). In the present study, both RF-IgA and RF-IgM were significantly elevated, suggesting polyclonal B cell activation; however, no significant difference was observed in RF-IgG, suggesting that different isotypes may have distinct clinical implications. Based on these data, we recommend that anti-CCP testing be incorporated into the routine evaluation of pSS patients, particularly for those with articular symptoms.

The most clinically striking finding of this study was that, despite having longer disease duration and more active inflammatory markers (elevated ESR and IL-6), the overlap group had a significantly lower rate of immunosuppressant use (3.39% vs. 23.60%, *p* = 0.001) and a significantly higher rate of biologic agent use (28.81% vs. 8.99%, *p* = 0.002), with no difference in glucocorticoid use (approximately 75% in both groups). This seemingly paradoxical treatment pattern requires multidimensional interpretation. First, conventional csDMARDs may have limitations in the overlap setting: methotrexate may worsen sicca symptoms and increase hepatotoxicity risk in pSS patients (34), and some pSS-RA patients may respond poorly to methotrexate (35). Second, a preference for biologic agents may be emerging: with the widespread use and improved accessibility of biologic agents in RA treatment, clinicians may be more inclined to choose biologic agents with well-defined mechanisms of action and rapid onset for overlap syndrome patients, particularly when there is a risk of joint erosion or failure/intolerance of conventional DMARDs. Third, there may be a match between disease immune characteristics and drug selection: the immunological findings of this study suggest that overlap group is primarily driven by the IL-6 pathway, which is not directly targeted by conventional csDMARDs but can be precisely targeted by IL-6 inhibitors. Furthermore, there are currently no specific treatment guidelines for the pSS-RA overlap syndrome (36). In the absence of high-quality evidence, treatment decisions by clinicians may vary considerably. In our cohort, clinicians tended to favour biologic agents over conventional csDMARDs in pSS-RA overlap patients, a pattern that may reflect the perceived need for more potent immunomodulation in the setting of elevated IL-6 and inflammatory burden. However, whether this approach confers superior efficacy remains unknown and requires rigorous evaluation in future prospective comparative studies.

This study has several clinical implications and innovative values. At the diagnostic level, the marked elevation of anti-CCP suggests that routine anti-CCP testing may facilitate early identification of concomitant RA in pSS patients, and an anti-CCP level >10 U/mL may serve as a screening threshold. At the mechanistic level, this study proposes an IL-6-driven hypothesis, distinguishing pSS-RA (predominantly IL-6/T cell axis) from pSS alone (predominantly B cell/interferon axis), providing a new perspective for understanding the pathophysiology of overlap syndromes. At the therapeutic level, the real-world treatment strategies observed in pSS-RA patients are consistent with their immune profile, offering a reference for clinical decision-making and a theoretical basis for the design of future clinical trials.

Based on the above findings, several directions for future research merit consideration. Prospective cohort studies with standardized disease activity assessments (DAS28-ESR and ESSDAI) are needed to validate the immune signatures observed in this study. Randomized controlled trials or comparative effectiveness studies are warranted to evaluate the efficacy and safety of biologic agents versus conventional csDMARDs in pSS-RA overlap patients. Mechanistic studies utilizing animal models or *in vitro* co-culture systems could further elucidate the role of the IL-6 pathway in the overlap phenotype. Additionally, multi-centre studies with larger sample sizes are required to confirm our findings and to establish the optimal anti-CCP cut-off value for screening concomitant RA in pSS patients.

Several limitations of this study should be acknowledged. First, the study was conducted in a pSS-centric cohort; therefore, we cannot establish directionality, and disease activity scores such as DAS28-ESR and ESSDAI were not available due to the retrospective design. We relied on ESR, cytokine levels, and imaging findings as proxies. Second, radiographic imaging for erosive changes was not performed uniformly across all patients, which may have led to underestimation of subclinical joint involvement in the pSS group. Third, although no patients received rituximab, exposure to other biologic agents may have influenced inflammatory profiles; we adjusted for biologic use in multivariable models, but residual confounding cannot be fully excluded. Fourth, all analyses were exploratory, and no adjustment for multiple comparisons was applied. Finally, the relatively small number of overlap patients limited detailed subgroup analyses. Despite these limitations, our findings provide real-world evidence and generate hypotheses for future studies.

In summary, in this pSS patient cohort, those with concomitant RA exhibit an immunological signature that closely aligns with RA immunopathology, characterized by IL-6 pathway activation, T cell activation, relative B cell suppression, and marked elevation of anti-CCP and RF. Clinically, these patients receive fewer conventional csDMARDs and more biologic agents. These findings suggest that RA is the most likely driver of the immune phenotype in the overlap syndrome, although causal direction cannot be established from this pSS-centric study design. Prospective studies enrolling RA patients are needed to validate causal inferences, and IL-6 represents a promising therapeutic target that warrants further investigation in this population.

## Data Availability

The original contributions presented in the study are included in the article/**Supplementary Material**. Further inquiries can be directed to the corresponding author.
