# Supplementary material for: Immunological profiling and treatment patterns of primary Sjögren’s disease patients with concomitant rheumatoid arthritis: a retrospective cohort study
Source: Front Immunol. 2026 Aug 3;17:1891805. doi: 10.3389/fimmu.2026.1891805 (PMC13478203; doi:10.3389/fimmu.2026.1891805)
Supplement: Supplementary file 1 [file Table1.doc]

Supplementary Table S1. Low-frequency autoantibodies

| Variable | pSS group(N=83) | pSS+RA group(N=56) | Statistic | P value |
| --- | --- | --- | --- | --- |
| Anti-Scl-70 positive, n (%) | 4 (4.82) | 3 (5.36) | Fisher | >0.999 |
| Anti-CENP-B positive, n (%) | 3 (3.61) | 1 (1.79) | Fisher | 0.648 |
| Anti-Sm positive, n (%) | 3 (3.61) | 4 (7.14) | Fisher | 0.439 |

Note: These antibodies were excluded from the main table due to low positive rates (<6%) and lack of significant intergroup differences.

**Table S2**. Complete comparison of absolute lymphocyte counts and all cytokines

| Variable | pSS（n=82）**†** | pSS+RA （n=56）**†** | U | value‡ |
| --- | --- | --- | --- | --- |
| Lymphocyte subsets (cells/μL) |  |  |  |  |
| CD3^+^T cells | 1133（809.0，1595） | 1153（791.5，1564） | U=2185 | 0.6322 |
| CD4^+^Th cells | 590.4（474.2，879.3） | 774.8（460.9，1082） | U=1905 | 0.0903 |
| CD8^+^ cells | 389.8（268.0，632.7） | 365.5（242.2，540.7） | U=2091 | 0.3759 |
| CD19^+^B cells | 229.5（174.6，356.9） | 191.4（103.3，300.8） | U=1867 | 0.0629 |
| CD16^＋^CD56^+^NK cells（%） | 10.57（7.560，16.00） | 13.04（8.368，18.77） | U=1886 | 0.0756 |
| CD16^＋^CD56^+^NK cells（cells/μl） | 167.8（96.68，277.0） | 183.4（117.8，339.8） | U=1897 | 0.0834 |
| T＋B+NK（%） | 99.22（98.70，99.52） | 99.49（99.12，99.63） | U=1814 | **0.0364** |
| T＋B+NK（cells/μl） | 1455（1132，2233） | 1602（1263，2211） | U=2096 | 0.3877 |
| Cytokines (pg/mL) |  |  |  |  |
| IL-6* | 1.580（0.5800，4.300） | 5.000（1.885，15.43） | U=1367 | **<0.0001** |
| IL-17* | 1.770（0.6100，2.140） | 1.770（1.770，4.030） | U=1722 | **0.0077** |
| IL-8* | 1.840（0.9200，2.000） | 2.000（1.730，2.053） | U=1811 | **0.0232** |
| TNF-α* | 0.2200（0.2200，1.440） | 0.4000（0.220，1.430） | U=2215 | 0.6324 |
| IL-1β(n=80/56)§ | 1.678（0.4200，3.750） | 2.695（0.8550，3.313） | U=1976 | 0.2436 |
| IL-10* | 0.8600（0.2700，2.300） | 0.8200（0.2700，1.634） | U=2317 | 0.9752 |
| INF-γ* | 2.240（0.4900，5.450） | 2.305（1.613，5.523） | U=2129 | 0.4037 |
| INF-α | 0.8000（0.7100，0.8000） | 0.8000（0.7200，1.260） | U=2219 | 0.6501 |
| IL-2* | 0.9200（0.3900，1.590） | 1.070（0.7675，1.665） | U=1965 | 0.1226 |
| IL-4* | 0.2400（0.2400，1.170） | 0.5600（0.2400，0.9175） | U=2044 | 0.2226 |
| IL-12P70 | 0.8600（0.8600，1.330） | 0.8300（0.8600，1.330） | U=2214 | 0.6306 |
| IL-5 | 0.5800（0.3400，1.500） | 0.5800（0.5800，1.500） | U=2229 | 0.6784 |

†Data are presented as M (Q₁, Q₃) (see Table 1 for definition). Absolute lymphocyte counts were available in 82 pSS patients and 56 pSS+RA patients.

‡P values were calculated using the Mann–Whitney U test for non-normally distributed continuous variables.

§Sample size differs from the overall cohort due to missing data: IL‑1β was missing in 9 pSS patients (not recorded in medical records). All other cytokines had available data in 83 pSS patients (due to additional testing) and 56 pSS+RA patients.

*These indicators (IL‑2, IL‑4, IL‑6, IL‑8, IL‑10, IL‑17, IFN‑γ, TNF‑α) are also presented in the main text (Table 4) and are shown here for completeness.

*Cytokine detection limits and imputation methods are described in the Methods (section 2.3).
